# The best encouraging persons in labor: A content analysis of Iranian mothers' experiences of labor support

**DOI:** 10.1371/journal.pone.0179702

**Published:** 2017-07-06

**Authors:** Tahereh Fathi Najafi, Robab Latifnejad Roudsari, Hossein Ebrahimipour

**Affiliations:** 1 PhD Student, Student Research Committee, Department of Midwifery, School of Nursing and Midwifery, Mashhad University of Medical Sciences, Mashhad, IR Iran; 2 Associated Professor, Evidence-Based Care Research Centre, Mashhad University of Medical Sciences, Mashhad, IR Iran; 3 Associate Professor in Health Services Management, Social Determinants of Health Research Center, Mashhad University of Medical Sciences, Mashhad, IR Iran; National Institute of Health, ITALY

## Abstract

**Background and aims:**

The process of giving birth is very stressing for the mother. Meanwhile, maternity ward staff’s lack of awareness of mothers’ fears make mothers feel lonely and helpless. This study aimed to explore women’s perceptions of labor support during vaginal delivery.

**Materials and methods:**

This exploratory qualitative study used qualitative content analysis to explore Iranian mothers’ experiences of labor support. Data were collected using observations and semi-structured interviews with 25 individuals. The participants were recruited through a purposive sampling method.

**Results:**

Three categories, including “involvement of the spouse in the labor process”, “asking for a companion during labor”, and “mother’s self-care to cope with labor pain”, emerged during data analysis. These categories were merged to form the main theme of “trying to comply with the labor process”.

**Conclusion:**

Women believed that the presence of a companion, e.g. their husband, a family member, or a doula, during labor helped them better deal with the labor process, particularly when they felt lonely. Health care providers are expected to consider the needs of mothers and try to provide holistic support for mothers during labor pain.

**Implications for practice:**

It seems that some mothers adopted particular coping strategies without receiving any relevant training. It is noteworthy that although mothers may make every effort to minimize their pain, health professionals should also practice medical approaches to help them through the process of labor.

## Introduction

Childbirth is a unique complex, multidimensional, mental-cognitive experience and a major crisis in a woman’s life [1, 2]. It has a variety of psychosocial and emotional aspects and creates memories, sometimes traumatic, which will always remain in the minds of mothers [3]. The high levels of stress mothers face during the childbirth will constantly involve their body and mind and make them experience a wide range of positive and negative emotions [4, 5]. Due to their fears and negative attitudes towards labor pain, pregnant women tend to ask for support and care from midwives and maternity ward staff in stressful and painful conditions of the delivery room. To a mother in labor, a midwife is somebody who has enough knowledge, knows the needs of women, and continuously seeks to increase the mother’s sense of participation by evoking positive feelings in her [5]. However, maternity ward staff’s lack of awareness of mothers’ fears and negative attitudes creates a sense of loneliness and helplessness in the mother [5]. Therefore, in order to prevent the feeling of helplessness in mothers and control the conditions of the delivery room, mothers should be supported during labor.

Following the changes in treatment approaches and family structure and the reduction in the admission time during the recent years, focus on supporting mothers during pregnancy, labor, and breastfeeding has increased [6]. During childbirth, a midwife can support the mother through various forms of non-pharmacological care including massaging and using the right words (physical support), reassuring the mother and emphasizing on her ability to do natural childbirth (information and emotional support), and adopting holistic approaches toward the clients and fully understanding them (social protection) [7].

Support has numerous effects which can be explained through two well-known models. According to Hoff Meyer (1991), two hypotheses, namely the buffer model and the main effect model, can describe continuous support [8–10]. The provided support, in all above-mentioned forms, leads to a balance between the neuroendocrine and psycho-immunological paths of the body and reduces the effects of secreted catecholamine followed by stress. The main effect model argues that continuous support stimulates the mentioned paths of the body. Based on the buffering model, body can control environmental stress. By supporting a mother in the delivery room, the midwife activates both models and minimizes the need for obstetric interventions. As a result of full support during labor, the mother will develop a sense of security. She will also feel that her dignity is respected and her privacy is protected [5]. Moreover, support acts against stress and affects the choice of a suitable strategy [11,12].

Studies have confirmed the beneficial effects of support provided by the midwife and caregiver [9, 12–16]. In a systematic review, Hodnett (2013) concluded that continuous support of the mother decreased the Cesarean section rates, shortened the length of labor, and reduction the need for analgesics [15]. A quantitative study in Iran showed that the continuous support provided by midwives during labor decreased the intensity of pain, length of labor, and incidence of postpartum depression [9, 14, 17–20]. Although numerous studies in different countries (including Iran) have reported the favorable effects of constant support given to the mothers in labor on the health of both the mother and the fetus, most of these studies adopted a positivist paradigm, and thus a quantitative approach, and mainly assessed the effects of continuous support on the process of childbirth. However, the support provided during labor can also create a positive attitude towards childbirth and turn the moments of pain into the most memorable moments of a woman’s life. Since no previous qualitative research in Iran has evaluated the role of support in physiological conditions such as labor, conducting a qualitative research in the sociocultural context of Iran may clarify the unexplored dimensions of labor support.

### Objective

This exploratory qualitative study aimed to explore Iranian mothers’ experiences of labor and labor support.

## Methodology

This study was a part of a doctoral dissertation which used qualitative content analysis to explore the process of Iranian mothers’ support. Content analysis is a systematic approach which provides new knowledge and insight into a particular phenomenon. It leads to valid inferences from data and is suitable for examining the experiences and views of people towards the issue of interest [21–23]. Iranian women who had recently given birth recruited in this study.Women were eligible if they aged over 18 (because of their better understanding of birth and marriage), lived in Mashhad, and were fluent in Farsi. A total of 25 women, including 16 women in labor and having the experience of a natural childbirth, two women who had recently given birth, one birth companion, one doula midwife, two midwives, one obstetrician, one resident in obstetrics, and one medical student, were finally selected. The husband of one of the participants, as well as a midwifery student and lecturer in midwifery were also included. Sample size in qualitative research depends on data saturation, which means that researchers reach a point in their analysis of data that sampling more data will not lead to more information related to their research questions. Researchers see in their data that a concept is mentioned frequently or described in similar ways by a number of people and this make them empirically confident that the descriptions of their categories are thick and saturated,. In this circumstance the researchers are allowed to stop sampling data and to refine their analysis [23]. We achieved saturation after interviewing with 25 people and no new concept or categories were developed thereafter.

Purposive sampling continued until data saturation occurred. In order to ensure maximum variation, we tried to select the participants who had maximum diversity in terms of age, education, work experience, and social class and were able to provide the greatest amount of information regarding support in labor and delivery rooms "Table 1".

**Table 1:** Characteristics of the participants.

Participant	Duration of interview	Working experience	Place of childbirth (Hospital)	Occupation and education	Pregnancy history	Age	Time past of delivery
**Participant1**	**38 minutes**	**—-**	Non-governmental hospital	Housewife-Associate Degree	Second pregnancy	27 years old	**6 months**
**Participant2**	40 minutes	**——**	Non-governmental hospital	Housewife-Associate Degree	Second pregnancy	22 years old	**20 days**
**Participant3**	45 minutes	**——**	social health-care hospitals	Housewife-Associate Degree	First pregnancy	32 years old	**25 days**
**Participant4**	61minutes	17 years	**——**	Midwife-Bachelor degree A Midwife-dula	**——**	35 years old	**———-**
**Participant5**	45 minutes	35 years	**——**	Obstetrician	**——-**	64 years old	**———-**
**Participant6**	50 minutes	5 years	**——**	PhD student of Petro chemistry **A gentleman**	**——-**	34 years old	**————**
**Participant7**	71 minutes	**——**	**——**	Midwifery student	**——-**	20 yearsold	**————**
**Participant8**	38 minutes	28 years	**——**	Midwife expert	**——**	54 years old	**————**
**Participant 9**	69 minutes	**——**	Non-governmental hospital	Bank employee-BC	First pregnancy	31 years old	**4 months**
**Participant 10**	59 minutes	**——**	Governmental hospital	Housewife-Student	First pregnancy	22 years old	**6 months**
**Participant 11**	114 minutes	**——**	social health-care hospitals	Housewife-Diploma	Second pregnancy	23 years old	**2 months**
**Participant 12**	79 minutes	**——**	Non-governmental hospital	Housewife-Student	First pregnancy	25 years old	**17 days**
**Participant 13**	74 minutes	6 years	Non-governmental hospital	School assistant**-BC**	First pregnancy	30 years old	**10 days**
**Participant 14**	48 minutes	**——**	Charity hospital	Housewife-Math BC	First pregnancy	28 years old	**40 days**
**Participant 15**	48 minutes	**——**	Governmental hospital	Housewife-Diploma	Second pregnancy	30 years old	**15 days**
**Participant 16**	92 minutes	4 years	Non-governmental hospital	Office worker-MA	First pregnancy	33 years old	**12 days**
**Participant 17**	76 minutes	**——**	Non-governmental hospital	Housewife-Diploma	First pregnancy	23 years old	**7 months**
**Participant 18**	75 Minutes	**——**	Governmental hospital	Housewife-Student	First pregnancy	34 years old	**6 months**
**Participant 19**	39 Minutes	30 years	**——**	Retired-Teacher **Participant's mother**	**——**	54 yearsold	**———**
**Participant 20**	70 minutes	**——-**	Non- Governmental hospital	Office worker-BS	First pregnancy	27 years old	**5 months**
**Participant 21**	40 minutes	**———**	Governmental hospital	Resident in Obstetrics	———	**28years**	**——-**
**Participant 22**	35 minutes	**——**	Governmental hospital	Medical student	———-	24 years	**———**
**Participant 23**	68 minutes	**——-**	Nursing and midwifery school	Lecturer of Midwifery	——-	29 years	**——-**
**Participant 24**	71 Minutes	**———**	social health-care hospitals	Housewife	First pregnancy	28 years	**5 months**
**Participant 25**	48 Minutes	**———**	Governmental hospital	Housewife- Bachelor of Phylosophy	Second pregnancy	35 years	**24 hours**

The sample size was increased to eliminate the possibility of fake saturation [24, 25]. The interviews were conducted in private and public health care centers and or at more convenient places, such as parks or the participants’ houses, at the participants’ request. Participants were selected from hospitals and health centers, The hospitals included four governmental hospitals, one social health-care hospital, three non-governmental (private) hospitals and two charity hospitals, members of websites, public places such as parks, and social networks like Telegram and Viber (by sending an invitation letter and an information sheet). Individuals who were interested in participation were then provided with details about the objectives of the study. Face-to-face semi-structured interviews were conducted to collect data. The interviews were started with a number of general questions such as “Would you please explain your experiences of labor?” or “Please tell me about the measures given by the labor ward staff to you at the time of labor.” A number of probing questions, e.g. “What do you mean exactly?”, were also asked to obtain clearer and deeper information. The interviews lasted between 38 and 114 minutes. Prior to the interviews, the participants were asked to provide informed consent for the conduct and recording of the interviews. Since five interviewees did not allow the recording, five interviews were not recorded.

The interviews were recorded using a digital recorder and transcribed verbatim shortly afterward. The participants were then provided with the transcripts and asked to confirm their accuracy. Field notes were taken before, during, and after the interviews in order to record the details of the interview context. Moreover, since observation is appropriate as a complementary method to collect data and explain the behaviors, the first researcher collected data through observation by spending 50 hours in the labor environment [23].Simultaneous with data collection, conventional content analysis, proposed by Graneheim and Lundman (2004), was adopted for data analysis [22]. Conventional content analysis uses a transparent and systematic eight-step process to extract codes and categories directly from the raw data. These steps included listening to all the interviews several times to gain general insight, transcribing the interviews verbatim, overviewing of the transcriptions to identify meaning units, reviewing the meaning units to code and categorize them, comparing the categories and sub-categories and classes, and ultimately extracting more abstract themes from the categorizes [21,26–27] "Table 2".

**Table 2:** The process of coding and identifying major categories.

Meaning Unit	Code	categories	Theme
**“I’m very glad that we're gonna have a baby again." He said, “I love you so much because you are going through all this pain because of me."p11**	Mother’s understanding of her husband's support	**Spouse involvement in the labor process**	**Trying to comply with the labor process**
**"When I got stuck in an bad event my housewife calm me effectively so I was trying to calm her with my verbally or holding her hands". P6**	Husband’s action to reduce the pain		
**"He always kisses my hand and says, “Thank you very much for giving me this son and creating the sense of parenting for me."(P 17)**	Efforts to finally become a father		
**" when I had a contraction I was worrying about my son because he was much more on pressure than me".p12**	Ebullition of a mother's love		
**"Honestly I had very good sisters, they turned to my side as I informed them. They support me effectively" (p 16)**	Presence of birth companion at the time of labor	**Asking for a birth companion during labor**	
**"I was surprised when I saw all these relatives. Well, this gives you reassurance that you are not alone.*"*.*(P 12)***	Expression of emotional feelings of relatives		
**"I was constantly looking at the timer at the time of pain and counting my pain".*(P 8)***	Self-learning of the mother during pain	**Mother's self care to cope with labor pain**	
**"The moments of labor is a special time you goanna to turn on GOD's bell and return". *P11***	The mother's understanding of the achievements of childbirth		
**"I am trying to relax own when my pain was started".*p20***	Self-caring of the mother during pain		
**"I encourage him and told that yes Okey pain is coming end so soon my son"…It's fulfillment my mother's love to baby" *P10***	Talking to the baby		
**"I concentrated to the honey moon days, when my housebound expressed his feeling". *P 9***	Distraction of mind and reminding the good days		

A variety of methods were used throughout the study to ensure the trustworthiness of the collected data. In order to verify the credibility of the data and codes, member check was performed to see if the extracted codes matched the participants’ views. For this purpose, the participants were provided with five drafts of the interviews and asked to verify if the researchers had actually presented their real perceptions and experiences. Moreover, peer debriefing was used to ensure credibility, i.e. the extracted codes and categories were reviewed by two expert supervisors and two PhD students. Moreover, allocation of sufficient time to the interviews, transcription of interviews immediately after their conduct, and simultaneous data collection and analysis enhanced the credibility of data. Furthermore, immersion, prolonged engagement in data collection and analysis, and providing field notes helped the trustworthiness of data.

### Ethical considerations

The study was approved by the Ethics Committee of Mashhad University of Medical Science (Mashhad, Iran) in 2014. Official permissions were also obtained from relevant authorities before entering the fields of study for both observation and interview. Moreover, all the participants signed an informed consent form after they had been provided with details on the study objectives and ensured about not only data confidentiality and anonymity, but also their right to withdraw from the study at any time (without their decision affecting their course of treatment).

## Findings

Overall, 1680 codes were extracted from the interviews (70–195 codes from each interview). After eliminating redundant codes, the number of codes was reduced to 1200. Afterward, similar codes were merged into primary categories which were then abstracted to three main categories including “involvement of the spouse in the labor process”, “asking for a birth companion during labor”, and “mother's self-care to cope with labor pain”. These categories formed the main theme of “trying to comply with the labor process” (Table 2).

### 1. Involvement of the spouse in the labor process

The presence of husbands during childbirth is accepted throughout the world. The involvement of the spouse during labor not only exerts some medical effects (e.g. decreasing the need for analgesics), but also promotes responsible parenthood and father-child bonding.Women in this study believed that their spouses could play a major role in reducing labor pain. Meanwhile, based on their characteristics, the spouses showed different reactions toward the stress they encountered. They even expressed feelings that they had never experienced.

“*He (my husband) was very encouraging and gave me a special energy*. *He was joking around a lot*. *He said it was nothing and it would pass*, *God willing*. *He said that the second and third deliveries would be easier*!”, recalled participants #12 (a 25-year-old housewife who had recently given birth).

Women are sensitive to any acts and words from their spouses. Therefore, any satisfying actions from the spouse’s side will have a positive effect on women. When experiencing labor pain, women need to receive emotional support through hear empowering words and being touched and kissed.

*“… He told me that he loved me so much because I was going through all that pain because of him*. *It was enough for me that he was holding my hand*. *He was telling me about the good days that we had together…*”, recalled participant #11 (a 23-year-old housewife who had given birth to her second child three years before the study).

Accompanying their wives through labor pain was a very distressing experience for men and evoked negatives emotions (such as fear of unknown consequences) among some fathers.

*“At first*, *he was stressed*, *but it was too short because he was frightened*, *too*. *When we were leaving*, *he told me not to panic*. *He really made me calm*.*”*, said participant #16 (a 33-year-old woman).

“*My husband was stressed when he realized that I was going to give birth*. *I’m usually very quiet when I am in pain*. *I didn’t say anything because I saw he was stressed*.*”*, reported participant #17 (a 23-year-old housewife).

### 2. Asking for a companion during labor

According to the participants, although maternity wards did not allow a companion in the delivery room, the presence of women’s husbands or mothers in the waiting room or being allowed to make a short contact with them could give women comfort and reassurance and made them feel loved.

“*During my first delivery*, *my father*, *my father-in-law*, *my mother*, *my sister-in-law*, *and everybody were praying for me behind the door of the delivery room*. *This makes you calm and relaxed*, *you know*, *when you think that there are people there for you*. *When you go in there (maternity hospital)*, *you feel very lonely*, *but when you hear them*, *it gives you a won*de*rful feeling*.*”*, commented participant #12 (a 25-year-old woman with a history of two deliveries).

Interestingly, the participants were not willing to have their mothers accompany them in the delivery room as they thought that their mothers would be so stressed seeing them suffering from labor pain.

*“I wanted my husband to be with me*, *because I knew that my mother would get anxious and suffer even more than me*. *I didn’t want her to be there at that moment*, *because I knew that she would panic*.*”*, said participant #1 (a 28-year-old housewife with a history of vaginal delivery).

“*All I worried about was that nobody was there for me*. *I mean all of it*, *that loneliness*, *it was very depressing*! *Although there was another patient in my room and I wasn’t alone in the room*, *I felt like there was nobody there*! *I had a very bad feeling*. *But I’m happy because of the moment of birth*. *I think that a baby is a fruit of love*, *the result of passion*. *I really wanted my husband to be there when I got out of that delivery room*, *but he wasn’t*.*”*, mentioned participant #18 (a 34-year-old woman who had delivered her first baby in a public hospital).

Such feelings of the participants were also confirmed by their mothers. In fact, the relatives present in the waiting room expressed overwhelming feelings. The mother of a participant (a 55-years-old retired teacher), who seemed very anxious when they brought her daughter to a private hospital for vaginal delivery, said:

*“I think*, *you know*, *someone who is going to give birth is so stressed herself and the other person who accompanies her is stressed*, *too*. *So I 'm afraid that my daughter gets more anxious when she sees my anxiety*.” (participant #19).

This scenario was a little different for husbands. Since they saw themselves supportive of their wives, they decided to return their wives’ kindness by acting like them during labor.

*“When I am upset about something*, *she usually puts her head on my shoulder and then I feel relaxed*. *I suppose that if I could be with her at these moments*, *it would be good for her*. *It would make her feel that somebody was supporting her and help her better manage her stress…*.*”*, stated participant #6 (a 35-year-old husband).

### 3. Mother’s self-care to cope with labor pain

The self-care and self-learning practiced by mothers during labor originated from their previous experiences or any information they obtained through regular trainings. In their attempts to cope with labor pain, our participants tried to adopt various strategies such as distracting themselves from labor pain, being prepared for the following contraction, measuring the duration of pain, using relaxation techniques during contractions, and believing in the superiority of the divine power over human abilities.

“*Honestly*, *when I arrived at the hospital*, *I knew that I had a tough job ahead*. *So*, *I tried to be calm and manage my stress*. *I kept telling myself that I had to pass this stage and that I had to do my best to overcome the difficulty of labor*.*”*, recalled participant #17 (a 23 year-old housewife who had just given birth to her first child).

“*It gives you a good feeling to talk to your baby when you’ve got pain*. *I suppose there is more pressure on my baby than me*, *because he moves in all directions in that small uterus*. *He is under more pressure*, *I think*. *So*, *I was more concerned about my baby than about myself*. *Another thing is that I counted my contractions as if I liked them*.*”*, said participant #11 (a 23-year-old woman) when she was discussing her method of dealing with labor pain.

However, in some cases, the pain became dominant and the mothers’ strategies were not effective. The mothers tried to find somebody to comfort or encourage them.

“*I always thought that I had to go through all these alone and that nobody would be there for me*. *This made me very upset*. *I thought that neither my mother nor my husband was allowed to be there*. *I wished that at least someone was there to understand what I said*, *to listen to me and to hear my groans*, *to understand me and reassure me that it was going to be over soon*. *I was looking for these words*! *These things could highly affect my confidence*.*”*, declared participant #12 (a 25-year-old woman) about her need for a supportive and reassuring companion during labor.

Another issue expressed by the mothers was their understanding of the achievements of labor and their expression of feelings at the moment of birth. Almost all mothers who gave birth through vaginal delivery expressed their feelings of pride and satisfaction. Although some participants had unpleasant feelings and experiences during labor, they were generally satisfied with their ability to do a great job.

*“I was happy*. *I was happy that I became a mother even though I knew it was a difficult responsibility*. *My baby was born*. *I was very happy*. *Everything seemed to be perfect and excellent*. *No problem came to my mind at that moment or maybe I’ve just forgotten my thoughts*.*”*, said participant #17 (a 23-year-old housewife).

As another strategy to deal with labor pain, mothers tried to concentrate on their babies and giving them their maternal love.

*“But I’m happy because of the moment of birth*. *I think that a baby is a fruit of love and the result of passion*. *I really wanted my husband to be there when I got out of that delivery room…”*, stated participant #18 (a 34-year-old primiparous woman).

When mothers’ strategies did not work due to the severity of pain, our participants sought a comforting and encouraging person.

*“I had always thought that I have to go through all these alone and nobody would be there for me*. *This made me very sad*. *I was looking for someone to tell me that it was nothing and that it would be over*! *These things could highly affect my confidence*.”, recalled participant #12 (a 25-year-old mother).

## Discussion

This exploratory qualitative study aimed to investigate mothers’ experiences of labor support. Three main categories, including the “involvement of the spouse in the labor process”, “asking for a companion during labor”, “mother’s self-care to cope with labor pain”, emerged during data analysis. These categories were then combined into a main theme: “trying to comply with the labor process”.

### Involvement of the spouse in the labor process

When a woman goes through labor pain, her husband can provide her with great support by discussing his feelings. Kharesheh et al. (2010) compared women who received support from their relatives during labor with a control group (women who were only accompanied by medical personnel). Although the length of labor was similar in the two groups, those supported by their family needed lower amounts of analgesics and had a more pleasant experience during labor [28]. In a clinical trial, McGrath (2008) showed that husband’s support at the time of labor not only reduced Cesarean section rates, but also produced other positive results in low-middle income countries [29]. In a systematic review, Hodnnet (2013) concluded that the support received from the relatives during labor decreased the length of labor, created good memories of labor in women’s minds, and reduced Cesarean section rates. No such effects were observed when women were only supported by professionals [15]. Receiving support during labor has also been reported to have positive effects on breastfeeding [28]. In addition, based on the results of previous qualitative studies, husband’s support during labor exerted positive effects on the relationship between the mother and the father and strengthened their bond with the baby [29, 30]. Similar results were also published in a meta-synthesis performed by Johansson (2015). Additionally, men’s involvement in the labor process was found to help them better move toward becoming a father [30]. Consistent with our findings, this meta-synthesis identified preparing for parenthood and providing appropriate support for the mother in the emotional challenging phase of labor as the most important themes [30]. Likewise, a qualitative study by Sapkota (2008) highlighted fathers’ “desire” and “curiosity” as the main reasons they wished to be present at labor to support their wives. In fact, rather than staying in the waiting room, fathers preferred to accompany their wives in the delivery room to support them and to share everything with them [31]. According to Escott et al., husbands adopted a variety of measures, such as verbal comforting, holding hands, and hugging, to help mothers and deal with labor pain [32]. The husbands used these measures at home before the women were transferred to the labor ward [32]. Although husbands are legally prohibited from attending the delivery room in Iran, most of our participants preferred to have their mothers and husbands on their side during labor. Most delivery centers in Iran are feminine somehow. Dearth of facilities and some certain religious issues in Iran has convinced women to accept this condition. Recently, as a result of changes in hospital managers’ awareness and attitudes, some hospitals have specified separate rooms for this purpose so that husbands can stay with their wives at the time of labor [33]. The family context of parenting has dramatically changed during the past two decades. One of the most obvious changes in this regard is the greater involvement of fathers in both child rearing and the childbirth process. Through their support for their wives during the process of labor, men take a significant step toward becoming a parent [32].

In this study, the husbands used calming and encouraging words and behaviors to help their wives relax during labor. Similarly, in their study of women’s approaches toward labor pain management, Klomp et al. (2014) identified three main themes, including preparation, support, and decision-making and control. Their participants highlighted the significance of the presence and support of their husbands, family members, or friends during labor. In fact, even if they did not talk, their presence helped mothers relax and regain their calmness. mothers could better deal with labor pain in the presences of their husbands or family members, and of course a professional health care provider [34,35].

Bäckström and Wahn (2011) performed a content analysis and emphasized the role of environmental conditions of the labor ward on fathers’ willingness to be engaged in the process of labor [36]. Such environmental factors were also found to affect husbands’ behaviors in this study. In fact, since husbands are not allowed in the delivery rooms in Iran, all their supporting measures were limited to the time before women entered the delivery room.

### Asking for a companion during labor

“Asking for a companion during labor” emerged as the second category in this study. Based on the World Health Organization’s recommendations, midwives should facilitate the presence of a companion during labor. However, it is important for the mothers to trust these companions and feel comfortable around them. Therefore, husbands, friends, and relatives are good choices [37]. In Iran, mothers’ care during labor is exclusively performed by the medical staff, e.g. midwives and obstetricians, who do not necessarily consider mothers’ emotional needs. Since almost all Iranian hospitals prohibit the presence of relatives in the maternity ward, most mothers feel lonely and seek a companion. Campero et al. (1998) showed that most Mexican women were alone because no other person was allowed to enter the maternity ward. Therefore, they preferred to have a doula [38]. In a clinical trial, Madi et al. (1999) found that the presence of the mothers’ partners decreased the need for medical interventions [39].

Diniz et al. (2014) emphasized the role of companions in preventing negative feelings in women and suggested that companionship during childbirth had to be accepted as a new policy in developing countries [40]. Although numerous studies have highlighted the significance of the presence of the mother’s partner or another trusted person during labor, this critical issue seems to be neglected by medical staff [41].

### Mother’s self-care to cope with labor pain

The findings of this study showed that mothers employed coping strategies like self-care and self-learning to deal with labor pain. Their practical strategies often originated from the maternal instinct or their previous experiences. Escott et al. (2004) explained that mothers used breathing and relaxation techniques and counted the duration of pain in order to cope with labor pain. Most women learned these strategies from their previous experiences [32]. Likewise, mothers in our study reported these strategies to effectively control their pain and cause better feelings. Spiby et al. (2003) showed that adopting such strategies helped mothers relax, control their emotions and fears, and decrease the intensity of pain [42]. According to Escott et al. (2011), depending on their previous experiences and also their physical and mental conditions, mothers adopted a variety of strategies to cope with the labor process. They concluded that the mother herself changed her strategy in different situations [32]. Our participants adopted various techniques, such as distracting themselves by thinking about good things and good days of life, measuring the length of pain, and expressing joy when the pain ended, to cope with labor pain. Similar strategies were also reported by Escott et al. (2011) [32].

While our participants had not received any training, they had developed employed some self- care strategies, such as talking to their baby, to cope with labor pain. Likewise, Escott et al. (2011) reported that in order to relax and reduce pain, mothers tried to talk to their babies during the pain. In addition, our participants considered labor pain to be purposeful, i.e. it led to the birth of the baby and development of a sense of motherhood. Therefore, they coped with it and described it as pleasant and enjoyable. Mothers in this study also used adaptation and using positive words and thoughts as other strategies to deal with pain. Moreover, they really longed to enjoy the presence of an encouraging person during labor. Similar findings were reported by Escott et al. [30,32].

Whitburn et al. (2014) indicated that mothers overcame their fears by mentally accepting the pain and preparing themselves for the next contraction. They concluded that women’s use of their own experiences of labor pain had an important role in their understanding of pain and led to better pain management [43]. Lally et al. (2008) argued that a positive attitude, believing in the purposefulness of labor pain, distracting the mind from the pain, and thinking about the good days could decrease the intensity of labor pain [44]. Similar strategies were also highlighted by our participants.

### Conclusion and implications

Women apply various strategies to cope with labor pain. Since they felt lonely in the delivery room, they were eager to have a companion during labor. However, despite the significant role of spouses, they were not allowed in the delivery room. Therefore, women had to develop strategies, some of which they had not learned before, to deal with labor pain. It is noteworthy that although mothers may make every effort to minimize their pain, health professionals should also practice medical approaches to help them through the process of labor. Moreover, the critical role of the husbands in calming women should be considered by the labor team, particularly midwives. Finally, health policymakers are expected to pay more attention to this issue and provide holistic support for mothers during labor pain.

This paper gives a deep insight about labor support in Iran’s clinical settings which is presented for the first time. It can promote a new vision for health care professionals and midwives to get familiar with basic preferences and needs of women in labor. Furthermore, it can provide a new chance for Iranian men to get more involved during labor and normal delivery. Also, it has developed a framework for some quantitative and qualitative studies that enrich our findings and contributes to knowledge.
